# Gangrenous appendicitis in Amyand’s hernia: Surgical approach under local anesthesia. Case report and review of the literature

**DOI:** 10.1016/j.ijscr.2021.01.048

**Published:** 2021-01-16

**Authors:** Giovambattista Caruso, Chiara Toscano, Giuseppe Evola, Salvatore Antonio Maria Benfatto, Martina Reina, Giuseppe Angelo Reina

**Affiliations:** a General Surgery Department, Santissimo Salvatore Hospital (ASP Catania), Paternò, Catania, Italy; b General and Emergency Surgery Department, Garibaldi Hospital, Catania, Italy

**Keywords:** Amyand’s hernia, Acute appendicitis, Local anesthesia, Case report

## Abstract

**Introduction and importance:**

Amyand hernia is a rare disease seen in approximatively 1% of all hernias, complications of it, like acute appendicitis, or perforated appendicitis are even more rare, about 0.1%. Its diagnosis is very difficult in the preoperative period: it is usually an incidental finding.

**Case presentation:**

We report an unusual case of perforated gangrenous appendicitis with peri-appendicular abscess occurring in an irreducible Amyand’s hernia. An 80-year-old male, with chronic obstructive disease and pulmonary emphysema, atrial fibrillation, acute myocardial infarction, underwent urgent surgery, under local anesthesia, for right incarcerated inguinoscrotal hernia.

He was found to have a perforated gangrenous appendicitis with peri-appendicular abscess within a right indirect inguinal hernia sac. Appendicectomy and Bassini’s hernia repair were performed under local anesthesia without any complications.

**Clinical discussion:**

The treatment of Amyand’s hernia is not standardized. The current generally accepted algorithm for Amyand’s hernia is essentially contingent on the appendix’s condition within the hernia sac.

**Conclusion:**

Appendectomy and primary herniorrhaphy, under local anesthesia, for type 3 of Amyand’s hernia, is a safe procedure and easy to perform and, if confirmed by further study, could be part of every surgeon’s knowledge.

## Highlights

• The Amyand’s hernia is a rare disease.
• Acute appendicitis in Amyand’s hernia is very very rare.
• Acute appendicitis diagnosis in Amyand’s hernia is very difficult in the preoperative period.
• The treatment of Amyand’s hernia is not standardized.
• Appendectomy and herniorrhaphy under local anesthesia is a safe procedure.

## 1 Introduction and importance

Inguinal hernia, defined as the protrusion of an organ or fascia through the wall of the containing cavity, is one of the most frequent surgical procedures followed by a surgeon. It is not unusual to find an incarcerated hernia (defined as the inability to reduce the hernia content); typically the hernia content is the omentum or small bowel [[1], [2], [3]].

In a very low frequency one can find the cecal appendix inside the hernia sac: this condition is called “Amyand’s Hernia” whether it is inflamed or not [3].

In 1735 C. Amyand described the first case of incarcerated inguinal hernia, containing a perforated appendix, in an 11-year-old boy [3].

Older studies claim that Amyand’s hernia occurs in 1% of all inguinal hernias, however, more recent research suggests that the prevalence is smaller than previous thought, occurring in 0.4–0.6% of all inguinal hernias [5].

The presence of appendicitis within an Amyand’s hernia accounts for 0.07–0.13% of all appendicitis [4].

It is often diagnosed during hernioplasty, more commonly in children because of a patent vaginal process, but there are also cases of Amyand’s hernia reported in the range from a neonatal period to 92 years old [2].

Literature review reports a perforated appendix in 0.1%, with mortality range from 15 to 30% because of severe abdominal sepsis.

The present work has been reported in accordance with the Surgical Case Reports (SCARE 2020) criteria [6].

## 2 Presentation of case

An 80-year-old male, heavy smoker, with a history of atrial fibrillation, acute myocardial infarction, chronic obstructive pulmonary disease with pulmonary emphysema, and a 15-year history of reducible right inguinoscrotal hernia presented to the emergency department on June 2020 with acute abdominal pain, right groin mass, with associated nausea, constipation, and abdominal distention. The patient reported antihypertensive, antiarrhythmic, oral anticoagulant and bronchodilator therapy. No family history of the same condition was documented.

Vital signs were blood pressure of 140/90 mmHg, HR: 110 bpm arrhythmic due to atrial fibrillation, respirations of 18 with oxygen saturation: 94%, and temperature of 37,4 °C.

**Fig. 1:**
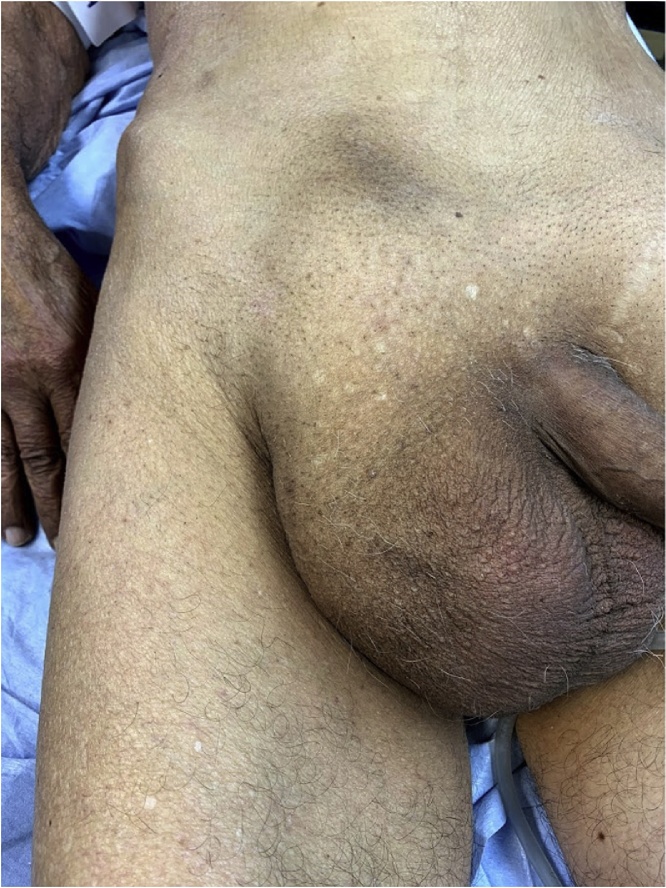
Clinical finding.

Examination revealed a soft abdomen with right lower quadrant tenderness to palpation, without Blumberg sign, and a nonreducible erythematous groin mass suggestive for incarcerated inguinal hernia (Fig. 1).

Blood testing showed leukocytosis of 10.200/mmc with a predominance of neutrophilis, CRP of 83.1 mg/l, INR: 2.13 (chronic oral anticoagulant treatment), SARS-COV2 nasopharyngeal swab negative.

Clinical diagnosis of right incarcerated inguinoscrotal hernia was clearly made and, after discussing the risks and benefits with the patient, he agreed to proceed with surgery and was carried to the operating room for an emergent right inguinal hernia exploration.

The preoperative ASA-physical status was 3–4 and considering the respiratory, cardiological and coagulative risks with contraindication to laparoscopic approach, the anesthesiologists proposed an open surgery under locoregional anesthesia. The surgery was performed by a surgeon experienced in emergency surgery and outpatient hernia surgery.

**Fig. 2:**
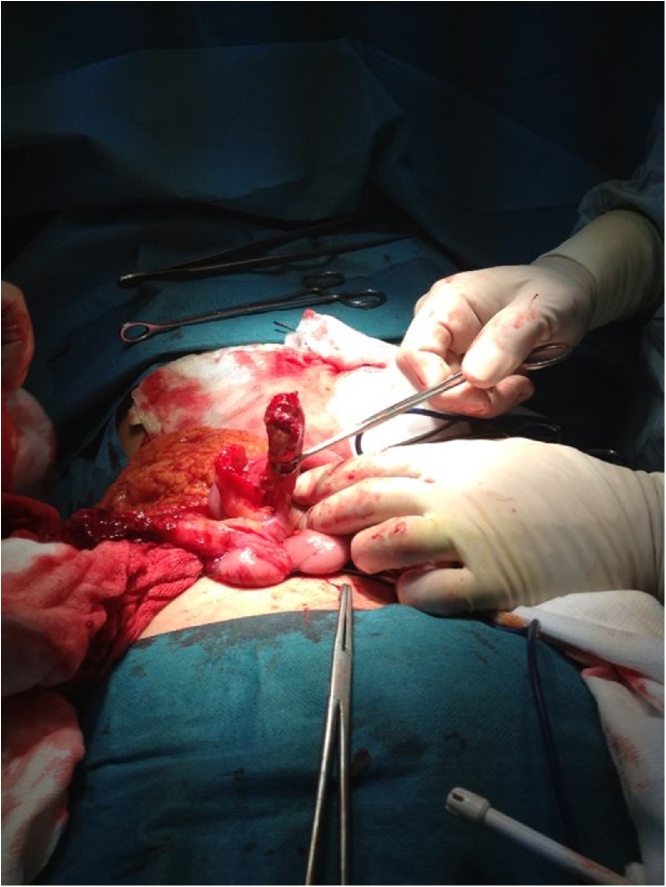
Intraoperative finding.

After infiltration of the line of incision with Lidocaine (20 mg/mL) 10 mL and the deeper planes with Levobupivacaine (5 mg/mL) 20 mL, we made a standard approach; when we opened the indirect hernia sac, encountered a perforated gangrenous appendicitis and an adjacent abscess (Fig. 2).

The abscess was drained, and the appendix was removed. After lavage of abdominal cavity and positioning of pelvic drainage, a Bassini hernia repair was then performed. A subcutaneous aspiration drainage was placed at the end of the procedure. The postoperative diagnosis was Amyand’s hernia with perforated gangrenous appendicitis. No intraoperative pain or discomfort was referred by the patient. Operating time was 40 min. No immediate postoperative complications were observed. The patient received a 24-h course of antibiotics and was discharged after 48 h. At follow-up, ten days, one month and six months after the surgery there were no wound complications or hernial recurrence.

## 3 Clinical discussion

Amyand’s hernia is very difficult to diagnose preoperatively. The differential diagnosis includes incarcerated or strangulated inguinal hernia, inguinal lymphadenitis, testicular torsion, acute epididymitis, acute hydrocele, and focal panniculitis [7].

The clinical presentation generally mimics that of an incarcerated inguinal hernia. The diagnosis is generally made at the operating room but in some cases the diagnosis is made preoperatively with the aid of inguinal echography and abdominal tomography [7].

The exact mechanism of appendicitis, within an inguinal hernia is not fully understood [8].

After a thorough review of the existing literature, some opinions are reported below:

1) Incarceration and, subsequently, inflammation of the appendix [9,10].
2) The presence of the appendix within the hernia sac predisposes for the development of adhesion between its serous membrane and the hernia sac, resulting in an irreducible hernia, susceptible to injury [8].
3) The contraction of anterolateral abdominal muscles leads to an increase in intra-abdominal pressure, causing compression and functional obstruction of the prolapsed appendix [11].
4) Inflammatory swelling of the appendix may be the beginning of a vicious cycle. Thus, Amyand’s hernia becomes irreducible, accentuating the swelling due to venous stasis and causing an impaired microcirculation of the appendix wall, resulting in bacterial overgrowth and translocation [12,13].

The current generally accepted treatment algorithm for Amyand’s hernia is essentially contingent on the appendix’s condition within the hernia sac [14].

There is no standardized treatment for Amyand’s hernia.

In 2007 Losanoff and Basson proposed an Amyand’s hernia classification system that can be useful for intraoperative decision making (see Table 1) [7].

**Table 1:** Losanoff and Basson classification of Amyand’s hernia.

Classification	Description	Management
Type 1	Normal appendix in an inguinal hernia	Hernia reduction, mesh replacement
Type 2	Acute appendicitis in an inguinal hernia with no abdominal sepsis	Appendectomy, primary no prosthetics hernia repair
Type 3	Acute appendicitis in an inguinal hernia with abdominal and abdominal wall sepsis	Laparotomy, appendectomy, and primary no prosthetics hernia repair
Type 4	Acute appendicitis in an inguinal hernia with abdominal concomitant pathology	Same as type 3 plus management of concomitant disease

Most of the authors in case of noninflamed appendix do not suggest removal. The transection of a fecal-containing organ in an otherwise clean procedure may increase morbidity and mortality from septic complications and, again, the surgical manipulation, during appendectomy, in the base of the caecum could increase the recurrence rate of the inguinal hernia, due to detachment in the deep inguinal ring [2,8].

However, some authors propose a prophylactic appendectomy for the risk of future appendicitis [5].

If an acute appendicitis is associated with Amyand’s hernia, the classical recommendation is to proceed with appendectomy and herniorrhaphy. The use of mesh in such a situation remains controversial. According to Losanoff’s classification in every case of appendectomy a mesh should not be used due to the risk of infection and septic complications.

The treatment of choice in these cases, also as reported by Sharma H et al. in the series of 18 consecutive cases of Amyand’s hernia, is appendectomy using Bassini or Shouldice repair, along with thorough abdominal and pelvic washouts to reduce septic complications [16].

However, there are documented reports of successful outcomes for hernias, identified as type 2 Amyand’s hernia, performing appendectomy and tension free repair with mesh, as reported by Shaban et al. [14].

Laparoscopic repair of Amyand’s hernia, with and without mesh placement, is increasingly reported in literature [15].

Appendectomy can-be performed under general, regional and local anesthesia but the anesthetic risks are least with the latter. Jebbin NJ, in 2007, reported that appendectomy under local anesthesia is safe and effective [17].

Sharma et al., in a prospective study of 165 patients with appendicitis, reported that appendectomy under local anesthesia is quick, cost-effective and carries little morbidity and can be used for all ages [18].

We report the case of an eighty-year-old man who arrived at the operating table diagnosed with a strangulated right inguinal hernia, who underwent surgery under regional anesthesia as we usually do with elderly patients and with concomitant pathologies that contraindicate general anesthesia.

During the operation, a perforated gangrenous appendicitis with inguinoscrotal abscess (Amyand's Type 3) is found when the hernial sac is opened. We proceeded with appendectomy, under local anesthesia, without any discomfort for the patient.

Similar to what Sharma H. ​​reported [16], at the clinical follow-up at 1 month and 6 months after surgery, our patient did not present wound complications or hernial recurrence.

According to Losanoff, prosthetic repair of the hernial defect was avoided by preferring a primary Bassini’s hernioplasty.

In 2008, Tisdale and Barwell reported a similar case of appendicitis, with a peri-appendicular abscess, in an irreducible Amyand’s hernia treated with appendectomy and sutured hernia repair performed under local anesthesia [19].

## 4 Conclusion

We provide a case report of a rare entity known as an Amyand’s hernia that presented an incarcerated that was diagnosed intraoperatively with a perforated gangrenous appendicitis, known as type 3, and treated with an appendectomy and primary herniorrhaphy, under local anesthesia, with a successful outcome.

This procedure, safe and easy to perform, if confirmed by further study, could be part of every surgeon’s knowledge.

## Declaration of Competing Interest

All the authors certify that there is no conflict of interest regarding the material discussed in the manuscript.

## Funding

All the authors declare that this research didn’t receive any specific grant from funding agencies in the public, commercial, or not-for-profit sectors.

## Ethical approval

Ethical approval has been exempted by our institution because this is a case report and no new studies or new techniques were carried out.

## Consent

Written informed consent was obtained from the patient for publication of this case report and accompanying images.

## Registration of research studies

Not Applicable.

## Guarantor

The guarantor for this case report is Giovambattista Caruso.

## Provenance and peer review

Not commissioned, externally peer-reviewed.

## CRediT authorship contribution statement

**Giovambattista Caruso:** Conceptualization, Writing - original draft, Writing - review & editing, Data curation. **Chiara Toscano:** Resources, Data curation. **Giuseppe Evola:** Methodology. **Salvatore Antonio Maria Benfatto:** Visualization. **Martina Reina:** Data curation. **Giuseppe Angelo Reina:** Supervision.
